# Supplementary material for: A Cretaceous Chafer Beetle (Coleoptera: Scarabaeidae) with Exaggerated Hind Legs—Insight from Comparative Functional Morphology into a Possible Spring Movement
Source: Biology (Basel). 2023 Feb 2;12(2):237. doi: 10.3390/biology12020237 (PMC9953289; doi:10.3390/biology12020237)
Supplement: Supplementary file 1 [file biology-12-00237-s001.zip › supplementary Figures .pdf]

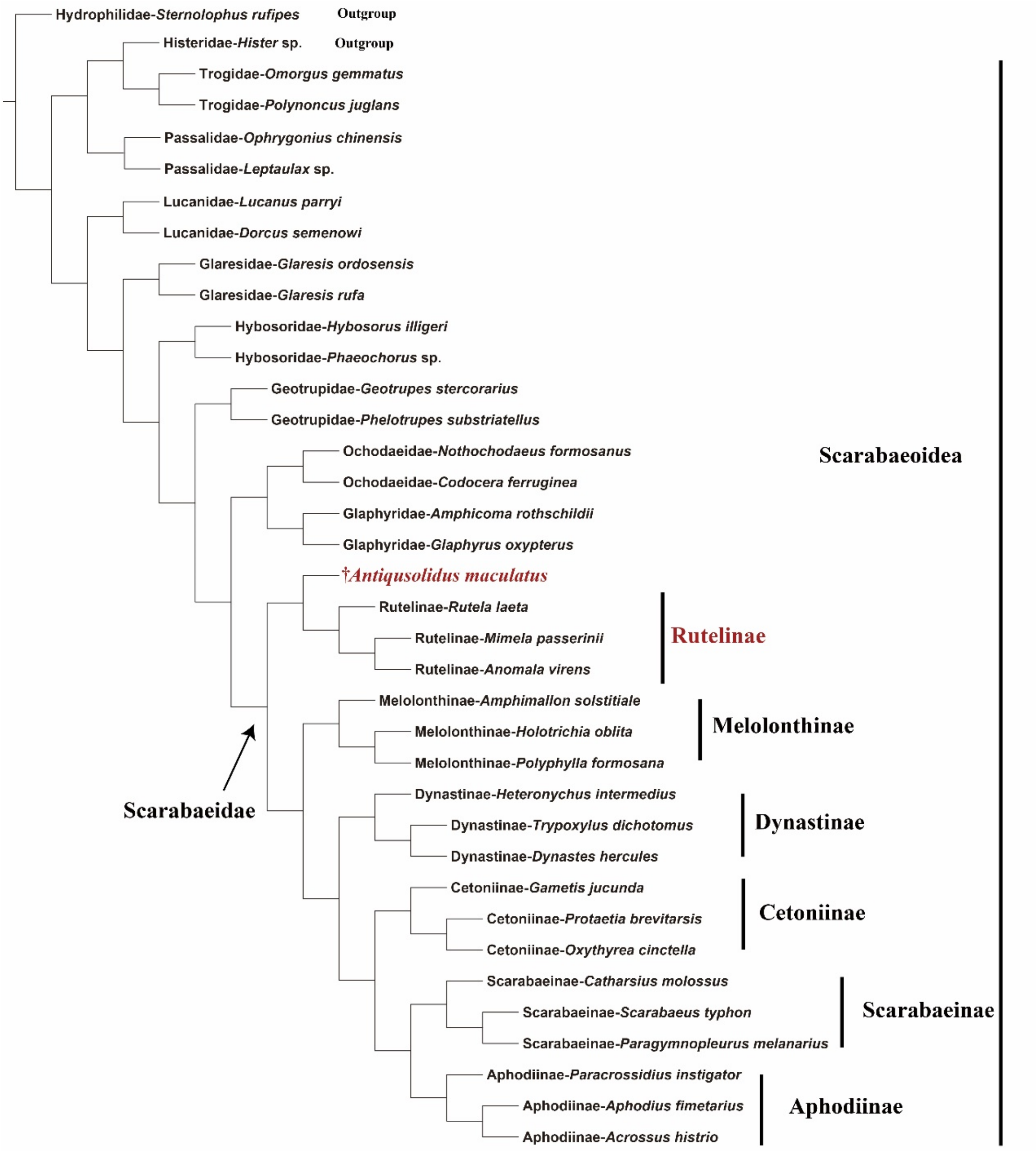

Figure S1. Strict consensus tree of 2 MP trees from equally weighted parsimony analysis using TNT.

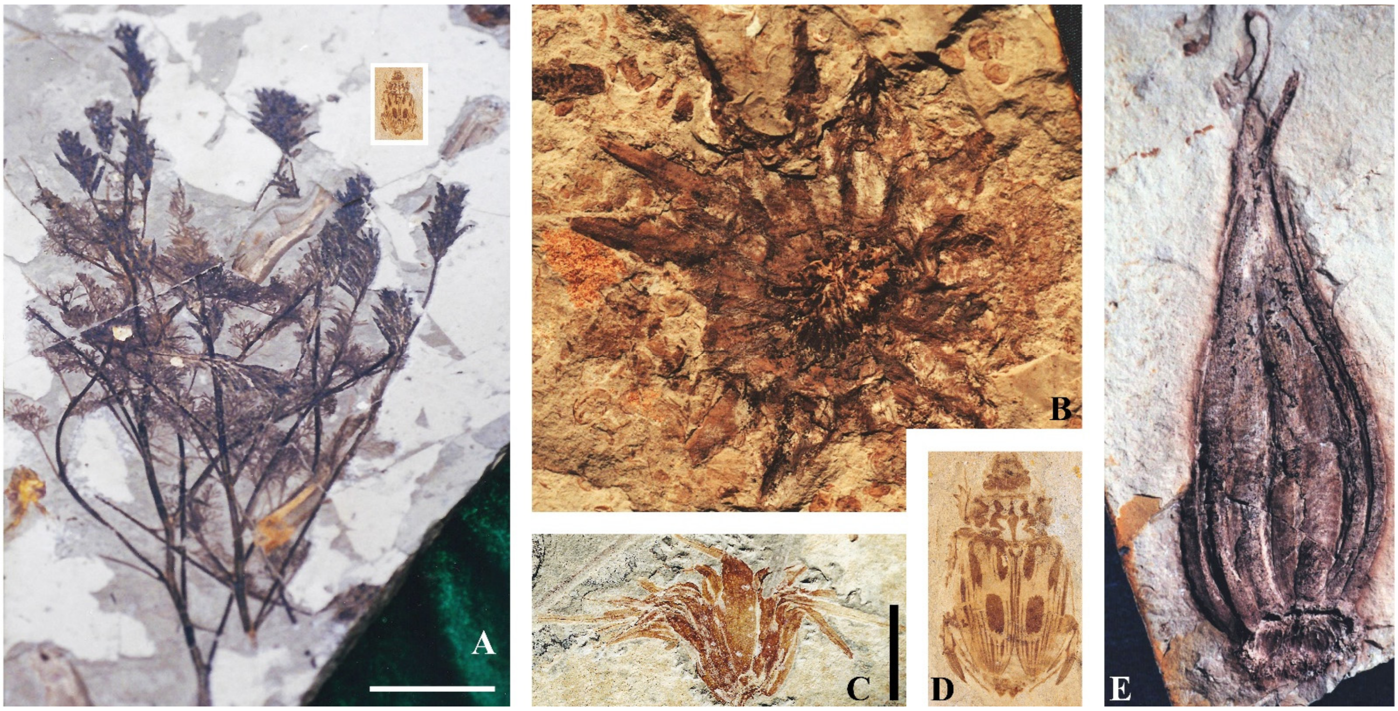

Figure S2. The size comparisons between *Antiqusolidus maculatus* and associated plants in Mesozoic. (**A**) *Archaefructus sinensis* Sun, Ji, Dilcher & Nixon, 2002 and *Antiqusolidus maculatus* Lu, Bai, Shih et Ren gen. et sp. nov. under the same scale, scale bar: 5 cm. (**B**) (**C**) (**E**) the reproductive organs of Bennettitalean plants (*Williamsonia* sp.) and (**D**) *Antiqusolidus maculatus* Lu, Bai, Shih et Ren gen. et sp. nov. under the same scale, scale bar: 10 mm.

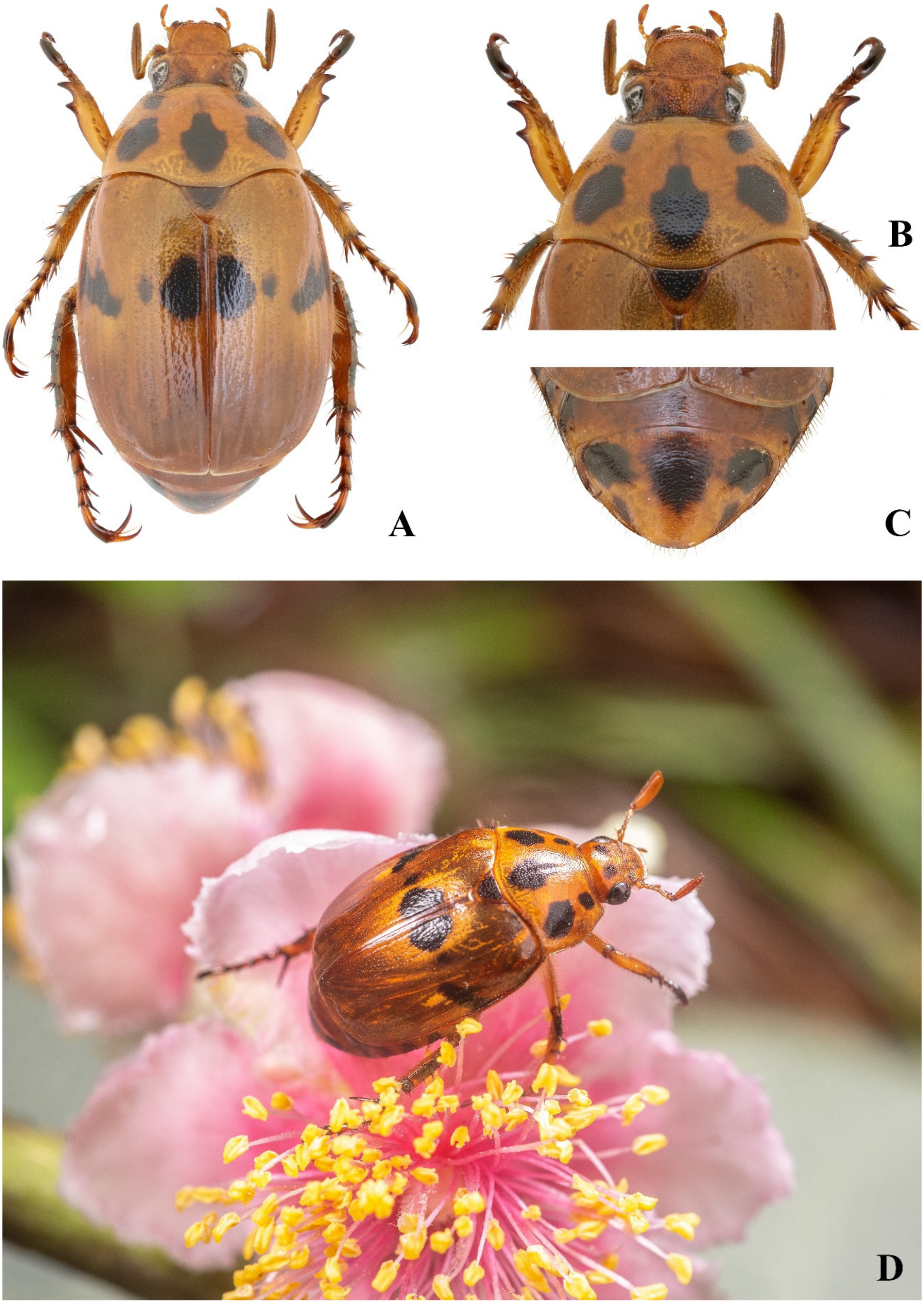

Figure S3. Markings and colour patterns for extant Rutelinae (*Anomala trivirgata* Fairmaire, 1888) feeding on flowers. (**A**) Dorsal view. (**B**) Head and Pronotum. (**C**) Pygidium. (**D**) Feeding on the flowers of *Actinidia eriantha*.
