# Supplementary material for: A Cretaceous Chafer Beetle (Coleoptera: Scarabaeidae) with Exaggerated Hind Legs—Insight from Comparative Functional Morphology into a Possible Spring Movement
Source: Biology (Basel). 2023 Feb 2;12(2):237. doi: 10.3390/biology12020237 (PMC9953289; doi:10.3390/biology12020237)
Supplement: Supplementary file 1 [file biology-12-00237-s001.zip › supplementary Table S2.pdf]

Table S1. A list of rutelines with marking patterns and feeding types.

Species	Marking patterns	Feeding types
Anisoplia agricola	Elytron: yellow and black markings	Feed on grass pollen and maturing grass seeds [1]
Anomala trivirgata	Pronotum, elytron, pygidium abdomen and legs: yellow with black markings	Feed on flowers of *Actinidia eriantha* (field observation, see figures S3D)
Leucothyreus marginaticollis	Elytron: brown with black markings in the middle	Feed on acerola leaves and flowers (*Malpighia emarginata*, Malpighiaceae) [2]
Parastasia gestroi	Yellow and black	Feed on and pollinate *Homalomena* sp. [3]
Parastasia nigripennis	Yellow and black	Feed on and pollinate *Homalomena* sp. [4]
Parastasia bimaculata	Yellow and black	Visit and pllinate inflorescences of *Homalomena propinqua* (Araceae) [4,5]
Mimela maculicollis	Yellow with black markings	Visit and pllinate the flowers of Dipterocarpaceae [6]
Proagopertha ohbayashii	Elytron: dark brown with yellow markings or yellow with black markings	Visit and pllinate the flower of *Leucaena leucocephala* [7]
Rutela lineola	Pronotum, elytron, pygidium abdomen and legs: dark brown with yellow markings	flowers of various plants [8]
Most of the species in tribe Anomalini	Simple color: green	Feed on leaves [9]
Most of the species in tribe Adoretini	Simple color: dull brown to yellow	normally nocturnal species [9]

Reference

1. Jameson, M.L.; Mico, E.; Galante, E. Evolution and phylogeny of the scarab subtribe Anisopliina (Coleoptera: Scarabaeidae: Rutelinae: Anomalini). *Syst. Entomol.* **2007**, *32*, 429–449. DOI: 10.1111/j.1365-3113.2006.00380.x
2. Ferreira, K.R.; Rodrigues, S.R. The mating behavior of Leucothyreus marginaticollis Blanchard, 1843 (Coleoptera: Scarabaeidae: Rutelinae). *Biota Neotropica.* **2017**, *17*, e20170330. http://dx.doi.org/10.1590/1676-0611-BN-2017-0330
3. Hoe, Y.C.; Gibernau, M.; Maia, A.C.D.; Wong, S.Y. Flowering mechanisms, pollination strategies and floral scent analyses of syntopically co-flowering Homalomena spp. (Araceae) on Borneo. *Plant Biol*. **2016**, *18*, 563–576. DOI: 10.1111/plb.12431
4. Kato, M.; Itioka, T.; Sakai, S.; Momose, K.; Yamane, S.; Hamid, A.A.; Inoue, T. Various population fluctuation patterns of light-attracted beetles in a tropical lowland dipterocarp forest in Sarawak. *Popul. Ecol.* **2000**, *42*, 97–104. DOI: 10.1007/s101440050014

5. Kumano, Y.; Yamaoka, R. Synchronization between temporal variation in heat generation, floral scents and pollinator arrival in the beetle-pollinated tropical Araceae Homalomena propinqua. *Plant Species Biol.* **2006**, *21*, 173–183. DOI: 10.1111/j.1442-1984.2006.00163.x
6. Kishimoto-Yamada, K.; Itioka, T. Seasonality in phytophagous scarabaeid (Melolonthinae and Rutelinae) abundances in an 'aseasonal' Bornean rainforest. *Insect Conserv. Divers.* **2013**, *6*, 179–188. DOI: 10.1111/j.1752-4598.2012.00201.x
7. Hirokawa, N. Records of Proagopertha ohbayashii (Coleoptera, Scarabaeidae, Rutelinae) found on the flower of Leucaena leucocephala in Okinawa Is., Okinawa Prefecture. *Saikaku Tsushin* **2010**, *20*, 45–46.
8. Calisto, V.; Morelli, E. Description of the immature stages of Rutela lineola (Linnaeus, 1767) (Coleoptera: Melolonthidae: Rutelinae). *Acta Zoológica Mexicana (nueva serie)* **2011**, *27*, 67–76.
9. Arrow, G.J. *The Fauna of British India, Including Ceylon and Burma. Coleoptera Lamellicornia part II (Rutelinae, Desmonycinae, and Euchirinae)*. Taylor & Francis: London, UK, 1917.
