# Supplementary material for: A Cretaceous Chafer Beetle (Coleoptera: Scarabaeidae) with Exaggerated Hind Legs—Insight from Comparative Functional Morphology into a Possible Spring Movement
Source: Biology (Basel). 2023 Feb 2;12(2):237. doi: 10.3390/biology12020237 (PMC9953289; doi:10.3390/biology12020237)
Supplement: Supplementary file 1 [file biology-12-00237-s001.zip › supplementary Text S1.pdf]

Text S1. List of characters used for the analysis of the phylogeny.

Body form:

1. Upper surfaces of body: (0) glabrous or subglabrous; (1) clothed with distinct hairs, setae or scales.

Head

2. Head at base: (0) declined less than 45 degrees; (1) declined at least 45 degrees.
3. Number of antennomeres: (0) 7; (1) 8; (2) 9; (3) 10; (4) 11.
4. Antennal modifications beginning on: (0) antennomere 4; (1) antennomere 5; (2) antennomere 5 or beyond.
5. Antenna: (0) not geniculate; (1) geniculate or elbowed.
6. Antennal insertion: (0) not visible from above; (1) visible from above.
7. Antennal club: (0) 3-segmented; (1) 4-segmented or more segments; (2) less than 3-segmented.
8. Antennal club: (0) loose; (1) compact.
9. Antennal club: (0) not lamellate; (1) lamellate.
10. Antennal club: (0) not cupuliform; (1) cupuliform.
11. Antennal club: (0) not or slightly flattened; (1) distinctly flattened.
12. Eye: (0) entire; (1) divided into upper and lower parts.
13. Frontoclypeal suture: (0) absent or incomplete; (1) indistinctly impressed; (2) distinctly impressed.
14. Anterior edge of clypeus or clypeolabrum: (0) straight to convex; (1) concave to shallowly emarginate; (2) deeply emarginate or excavate.
15. Mandible: (0) enclosed in mouth cavity or not visible in lateral view; (1) visible in lateral view.
16. Mandibles: (0) Visible from above; (1) not visible from above.
17. Mandibles: (0) Inner margin with few teeth or mandibles not greatly modified; (1) Inner edge serrate or mandible enlarged, elongated.

Pronotum

18. Prothorax widest: (0) anteriorly; (1) at middle; (2) posteriorly.
19. Lateral pronotal carinae: (0) visible for their entire lengths from above; (1) not visible for their entire length from above.
20. Anterior angles of pronotum: (0) absent, right or rounded, not produced; (1) produced and broadly rounded or obtusely angulate; (2) produced and narrowly rounded or acute.
21. Posterior angles of pronotum: (0) absent or broadly rounded; (1) obtuse or right; (2) moderately to strongly acute.
22. Pronotum: (0) without median longitudinal groove or line; (1) with median longitudinal groove or line.
23. Anterior edge of prosternum: (0) not produced anteriorly; (1) distinctly produced forming chin piece.

Scutellum

24. Scutellum: (0) well developed; (1) highly reduced; (2) absent or not visible.
25. Scutellum: (0) not abruptly elevated; (1) abruptly elevated.
26. Scutellum posteriorly: (0) narrowly rounded or acute; (1) broadly rounded or obtusely angulate; (2)

truncate.

Elytra

27. Abdominal tergites exposed by elytra: (0) none or apex of 1; (1) most of one; (2) at least one but less than 2; (3) at least 2 but less than 3.
28. Elytral apices: (0) meeting or almost meeting at the suture; (1) independently rounded or acute and separated by broad gap.
29. Epipleuron: (0) absent or incomplete; (1) complete.

Venter

30. Prosternal process: (0) complete; (1) complete, but interrupted, or imcomplete.
31. Median keel of mesonotum (medial view): (0) present; (1) absent.
32. Metaventrite: (0) flat to slightly convex; (1) moderately to strongly convex.

Legs

33. Procoxal cavities externally: (0) open; (1) closed.
34. Procoxal cavities externally: (0) narrowly closed; (1) broadly closed.
35. Mesocoxal cavities at middle: (0) contiguous; (1) narrowly separated; (2) moderately to widely separated.
36. Mesocoxal cavities: (0) strongly transverse; (1) circular to slightly transverse.
37. Mesocoxal cavities: (0) not or slightly oblique; (1) moderately to strongly oblique.
38. Metacoxae: (0) extending laterally to meet elytra or sides of body; (1) not extending laterally to meet elytra or sides of body.
39. Metafemur: (0) not much wider than mesofemur; (1) much wider than mesofemur.
40. Outer edge of protibia: (0) simple and rounded to carinate but without lobes or teeth, except at apex; (1) with one or more distinct lobes or teeth.
41. Mesotibia: (0) not strongly widened; (1) strongly widened, widest at or near apex.
42. Preapical surfaces of mesotibia: (0) without ridges or combs; (1) with transverse or oblique ridges or combs.
43. Metatibial spurs: (0) double; (1) single.
44. Metatibial spurs: (0) subequal in length and form; (1) differing distinctly in length; (2) differing distinctly in form.
45. Preapical surfaces of metatibia: (0) without ridges or combs; (1) with transverse or oblique ridges or combs.
46. Metatibial articular area: (0) not to only moderately expanded, narrowly oval or oblique; (1) greatly expanded, broadly oval to circular and flattened.
47. Mesotarsal paired claws: (0) subequal in length and similar in form and angle of inclination; (1) differing in length, or differing in form, or differing in angle of inclination.
48. Mesotarsal claws: (0) simple; (1) toothed or bifid; (2) serrate, denticulate or pectinate.

Abdomen

49. Number of abdominal visible ventrites: (0) 5; (1) 6; (2) 7.
50. Pygidium (sclerotized tergite 7 or 8): (0) more or less horizontal; (1) moderately to strongly oblique; (2) vertical or deflexed.

51. Number of functional spiracles: (0) eight; (1) seven; (2) less than seven.
52. 1-7 functional spiracles all situated in pleural membrane: (0) yes, (1) no.
53. 8th functional spiracles situated in pleural membrane: (0) yes, (1) no.
