# Supplementary material for: A Cretaceous Chafer Beetle (Coleoptera: Scarabaeidae) with Exaggerated Hind Legs—Insight from Comparative Functional Morphology into a Possible Spring Movement
Source: Biology (Basel). 2023 Feb 2;12(2):237. doi: 10.3390/biology12020237 (PMC9953289; doi:10.3390/biology12020237)
Supplement: Supplementary file 1 [file biology-12-00237-s001.zip › supplementary Text S2.pdf]

Test S2. Morphological matrix used for the analysis of the phylogeny.

Characters / Taxa		00000000011111111112222222222333333333344444444445555<br>12345678901234567890123456789012345678901234567890123
Hydrophilidae	*Sternolophus rufipes*	00220000000100001021000000100001000120000000010000000000
Histeridae	*Hister* sp.	0142101100001010120100111030001000210101101-0000012--
Geotrupidae	*Geotrupes stercorarius*	00420001110120100200010011000010121000111100100012001
Geotrupidae	*Phelotrupes substriatellus*	00420001110120100200000011000010121000111100100012001
Glaphyridae	*Amphicoma rothschildii*	10320001100020100100010001210010121000111000010011001
Glaphyridae	*Glaphyrus oxypterus*	10320001110020100100010001310010122000111101110011001
Glaresidae	*Glaresis ordosensis*	10320001110001100200200000000010121000111100010000000
Glaresidae	*Glaresis rufa*	10320001110001100200200000000010121000111100010000000
Hybosoridae	*Hybosorus illigeri*	00320001110000100202000000000010121010111100110011001
Hybosoridae	*Phaeochorus* sp.	00320001110000100202000000000010121010111000010011001
Lucanidae	*Lucanus parryi*	00321010101000101101000011000010121000010100100000000
Lucanidae	*Dorcus semenowi*	00321000101000101101000011000010121000010100100000000
Ochodaeidae	*Nothochodaeus formosanus*	10320001110010101111000010200011122000011001000011001
Ochodaeidae	*Codocera ferruginea*	10320001110010101111000011100011121000011101100011001
Passalidae	*Ophrygonius chinensis*	00320010111001100200011 2--000010121100010001000001 11-
Passalidae	*Leptaulax* sp.	0032000011100110020001 12--0000101211000100010000011 1-
Trogidae	*Omorgus gemmatus*	10320001100000110101100111000010120100011101100001110

Taxa \ Characters		00000000011111111112222222222333333333344444444445555<br>12345678901234567890123456789012345678901234567890123
Trogidae	*Polynoncus juglans*	10320001100000110101100111000010120100011001000001111-
Aphodiinae	*Aphodius fimetarius*	0022000110000001010100001000001012101001110111001111-
Aphodiinae	*Acrossus histrio*	0022000110000001010100001000001012101001110111001111-
Aphodiinae	*Paracrossidius instigator*	0022000110000001010100001000001012101001110111001111-
Scarabaeinae	*Scarabaeus typhon*	0022000111010201010000000--2000111220100101 1-00001211-
Scarabaeinae	*Catharsius molossus*	0022000111000001010000000--20001112201001111-11001211-
Scarabaeinae	*Paragymnopleurus melanarius*	0022000111010201010000000--20001112201001011-00001211-
Melolonthinae	*Holotrichia oblita*	0032000110002011010010001120001012100001010111011110-
Melolonthinae	*Polyphylla formosana*	0030001110002011010010001120001012100001010111011110-
Melolonthinae	*Amphimallon solstitiale*	1022000110002011010000001120001012100001010111001110-
Cetoniinae	*Protaetia brevitarsis*	0032010110000101020000000002010101220001111001100121 0-
Cetoniinae	*Oxythyrea cinctella*	00320101100001010200000000020101012200011110111001210-
Cetoniinae	*Gametis jucunda*	00320101100001010200000000020101012200011110111001210-
Dynastinae	*Trypoxylus dichotomus*	00320001100002110201000011100010121000111100100012 10-
Dynastinae	*Heteronychus intermedius*	00320001100002110201000011200010121000111101100012 10-
Dynastinae	*Dynastes hercules*	00320001100002110201000010100010121000111100100012 10-
Rutelinae	*Mimela passerinii*	0022000110002010010100001120001012100011110011111110-
Rutelinae	*Rutela laeta*	0032000110000210010110000120001012100011110111111110-
Rutelinae	*Anomala virens*	0022000110002010020100001120001012100011110011111110-
Fossil	*Antiqusolidus maculatus*	00???00110?0201001010000?12?001???10?01111001??01????
